# No Silver Bullet – Canonical Poly(ADP-Ribose) Polymerases (PARPs) Are No Universal Factors of Abiotic and Biotic Stress Resistance of *Arabidopsis thaliana*

**DOI:** 10.3389/fpls.2017.00059

**Published:** 2017-02-06

**Authors:** Dagmar Rissel, Peter P. Heym, Kathrin Thor, Wolfgang Brandt, Ludger A. Wessjohann, Edgar Peiter

**Affiliations:** ^1^Plant Nutrition Laboratory, Institute of Agricultural and Nutritional Sciences, Faculty of Natural Sciences III, Martin Luther University Halle-WittenbergHalle (Saale), Germany; ^2^Agrochemisches Institut Piesteritz e.V.Lutherstadt Wittenberg, Germany; ^3^Department of Bioorganic Chemistry, Leibniz Institute of Plant BiochemistryHalle (Saale), Germany

**Keywords:** abiotic stress, drought stress, flg22, plant immunity, pharmacological inhibition, poly(ADP-ribose) polymerases, salt stress, SRO proteins

## Abstract

Abiotic and biotic stress can have a detrimental impact on plant growth and productivity. Hence, there is a substantial demand for key factors of stress responses to improve yield stability of crops. Members of the poly(ADP-ribose)polymerase (PARP) protein family, which post-translationally modify (PARylate) nuclear proteins, have been suggested as such universal determinants of plant stress responses. A role under abiotic stress has been inferred from studies in which a genetic or, more commonly, pharmacological inhibition of PARP activity improved the performance of stressed plants. To further elucidate the role of PARP proteins under stress, T-DNA knockout mutants for the three Arabidopsis thaliana PARP genes were subjected to drought, osmotic, salt, and oxidative stress. To exclude a functional redundancy, which was indicated by a transcriptional upregulation of the remaining parp genes, a parp triple mutant was generated. Surprisingly, parp mutant plants did not differ from wild type plants in any of these stress experiments, independent from the number of PARP genes mutated. The parp triple mutant was also analyzed for callose formation in response to the pathogenassociated molecular pattern flg22. Unexpectedly, callose formation was unaltered in the mutant, albeit pharmacological PARP inhibition robustly blocked this immune response, confirming previous reports. Evidently, pharmacological inhibition appears to be more robust than the abolition of all PARP genes, indicating the presence of so-far undescribed proteins with PARP activity. This was supported by the finding that protein PARylation was not absent, but even increased in the parp triple mutant. Candidates for novel PARP-inhibitor targets may be found in the SRO protein family. These proteins harbor a catalytic PARP-like domain and are centrally involved in stress responses. Molecular modeling analyses, employing animal PARPs as templates, indeed indicated a capability of the SRO proteins RCD1 and SRO1 to bind nicotinamide-derived inhibitors. Collectively, the results of our study suggest that the stress-related phenotypes of *parp* mutants are highly conditional, and they call for a reconsideration of PARP inhibitor studies. In the context of this study, we also propose a unifying nomenclature of *PARP* genes and *parp* mutants, which is currently highly inconsistent and redundant.

## Introduction

The frequency and severity of abiotic stress conditions, such as drought or heat waves, are prospected to increase markedly in the near future due to the prevalent climate change. These incidences, which also exacerbate disease pressure, are difficult to predict and can occur during sensitive stages of the cropping season, with a potentially detrimental impact on crop yield. To safeguard crop productivity and food security, it is necessary to find ways to improve the plants’ performance under such conditions in the field. For this reason, there has been an intense search for key regulators in the plant’s genetic set-up that have robust and consistent effects on stress tolerance. Members of the Poly(ADP-Ribose) Polymerase (PARP) protein family *sensu stricto* have been presumed to possess this property, and the interference with PARP activity -pharmacologically or genetically- has been suggested to improve plant stress responses (De Block et al., 2005; Jansen et al., 2009; Geissler and Wessjohann, 2011; Schulz et al., 2012).

Proteins of the PARP family are present in all eukaryotes except yeast. They are characterized by a PARP domain (Karlberg et al., 2013). The best-studied member of this protein family is its founding member human PARP1 (HsPARP1). Activated upon DNA strand breaks, HsPARP1 forms poly(ADP-ribose) chains by attaching ADP-ribose molecules to nuclear proteins, including itself, using NAD^+^ as substrate. This fast and transient protein modification activates the DNA repair machinery (Pines et al., 2013). In humans, the PARP family comprises 17 members of which not all have PARP activity (Karlberg et al., 2013; Pines et al., 2013). In the model plant *Arabidopsis thaliana* three canonical PARP proteins have been identified, PARP1, PARP2, and PARP3 (Lepiniec et al., 1995; Babiychuk et al., 1998; Doucet-Chabeaud et al., 2001; Hunt et al., 2004). Unfortunately, the nomenclature of those Arabidopsis PARP proteins has been inconsistent in the past, with PARP1 and PARP2 being interchanged (Supplementary Table 1). In the following, PARP1 stands for the protein with the highest similarity to HsPARP1, encoded by At2g31320, while PARP2 is the protein encoded by At4g02390. Similar to the inconsistent gene nomenclature, the denomination of mutants of those genes is currently redundant and not co-ordinated. In this paper, we propose a unified mutant nomenclature, as described in the “Results” section.

Similar to their human counterparts, Arabidopsis PARP proteins play a role in DNA damage responses and the maintenance of DNA integrity under a range of circumstances. Thus, they mediate DNA repair, but also trigger programmed cell death, in response to oxidative genome stress (Amor et al., 1998), and the expression of *PARP1* and *PARP2* is induced by ionizing radiation (Doucet-Chabeaud et al., 2001). Consequently, knockout mutants for both genes are hypersensitive to DNA-damaging agents (Jia et al., 2013; Boltz et al., 2014; Song et al., 2015; Zhang et al., 2015). Both proteins have been shown to be associated with chromatin (Babiychuk et al., 2001) and to be involved in an alternative non-homologous DNA end joining pathway (Jia et al., 2013). Poly(ADP-ribosyl)ating activity of PARP1 and PARP2 has been demonstrated, confirming the presumed enzymatic action of the proteins (Babiychuk et al., 1998; Feng et al., 2015). Thereby, PARP2 was found to be the main contributor to PARP activity in plants.

Aside from their positive role in DNA repair, early inhibitor experiments indicated an involvement of PARPs in oxidative stress responses (Berglund et al., 1996). This association was also apparent in experiments with *Brassica napus* calli, in which chemical PARP inhibition improved growth under oxidative stress (De Block et al., 2005). In the same study, knockdown of *PARP* gene expression in Arabidopsis by RNAi constructs led to an increased tolerance to methyl viologen (paraquat). Those transgenic lines also showed an improved performance under drought stress (De Block et al., 2005). This obviously negative effect of PARPs on abiotic stress tolerance was explained by the load of NAD^+^-consuming PARP activity on the plant’s energy status. Alternatively, transcriptome analyses indicated that *PARP* effects on stress tolerance may be due to an interference in transcriptional and hormonal responses (Vanderauwera et al., 2007). In that study, high-light stress trigged decreased transcriptional oxidative stress responses, but increased levels of abscisic acid (ABA) and ABA-responsive gene expression, in *PARP1* RNAi plants as compared to the wild type. Chemical PARP inhibition similarly improved growth under stress, but also under control conditions (Schulz et al., 2012).

Besides those reports on a likely involvement of PARPs in abiotic stress responses, there is evidence that this protein modification also interferes with pathogen responses. The bacterial peptides flg22 and elf18 trigger cellular signaling networks that eventually lead to the launch of defense responses, such as the deposition of callose or lignin and the accumulation of pigments. These stress responses were blocked in Arabidopsis seedlings treated with PARP inhibitors (Adams-Phillips et al., 2008, 2010). In addition, *parp1 parp2* double mutants were slightly more susceptible to Pseudomonas bacteria (Feng et al., 2015).

In addition to the three canonical PARP proteins, members of another protein family, SRO (Similar to RCD One), also contain the catalytic core of the PARP domain, but not the regulatory PARP domain (Jaspers et al., 2010b). This family comprises its founding member RCD1 (Radical-induced Cell Death 1) and its homologs SRO1 through SRO5. So far, RCD1 and SRO1 have been functionally characterized most extensively. RCD1 has initially been identified as a positive regulator of the tolerance to ozone and apoplastic superoxide, and *rcd1* mutants are hypersensitive to those stresses (Overmyer et al., 2000). Conversely, *rcd1* mutants are more resistant to methyl viologen, which triggers chloroplastic superoxide generation (Ahlfors et al., 2004; Fujibe et al., 2004). They are also more tolerant to freezing and UV-B radiation (Fujibe et al., 2004), but less salt-tolerant, which has been related to its interaction with the Na^+^/H^+^-antiporter SOS1 (Katiyar-Agarwal et al., 2006). The homeostasis of hormone signaling pathways, such as ABA, ethylene, salicylic acid, and jasmonate, is altered in *rcd1* mutants, and hence, RCD1 has been suggested to function as integrative node in hormonal signaling networks (Ahlfors et al., 2004; Overmyer et al., 2005). RCD1 interacts with numerous other proteins, many of which are transcription factors involved in stress responses (Jaspers et al., 2009; Vainonen et al., 2012). The protein most closely related to RCD1, SRO1, has partially redundant functions to RCD1 in development and stress responses (Jaspers et al., 2009; Teotia and Lamb, 2009). Taken together, SRO proteins are centrally involved in stress responses, redox regulation, hormonal signaling, and transcriptional networks.

All hitherto analyzed PARP-domain proteins (i.e., PARPs and SROs) have been suggested to act in various stress responses, whereby their involvement in different types of oxidative stress has been studied most extensively. In this context, SROs have positive or negative effects, depending on the nature of the stress. In contrast, canonical PARPs have been suggested as generally negative factors of abiotic stress tolerance, either by posing a load on energy status or by affecting transcriptional stress responses. However, there is only a very limited number of studies in support of such an effect of canonical PARPs, most of them based on pharmacological inhibition, which of course may not be selective to PARP targets but may also affect other proteins not looked at in these studies. Importantly, the degree of functional redundancy of the three *PARP* genes in stress responses is largely unclear. For this reason, we analyzed the response of Arabidopsis single, double, and triple *parp* knockout lines to various abiotic stresses and to a biotic cue. Surprisingly, in contrast to previous reports, plant performance was not altered in any of the mutant lines. Protein homology modeling indicated that the previously reported interferences of PARP inhibitors in responses to abiotic and biotic stress may have been caused by off-site effects on SRO family proteins. Such a more complex picture was supported by our finding that the knockout of all *PARP* genes leads to a constitutive activation of cellular PARP activity, possibly mediated by SRO proteins.

## Materials and Methods

### Plant Material

*Arabidopsis thaliana* T-DNA insertional mutant lines for *PARP1* and *PARP2* were obtained from Nottingham Arabidopsis Stock Centre (NASC) and are shown in Supplementary Table 2. The lines are part of the GABI-Kat (Rosso et al., 2003) and SAIL (Sessions et al., 2002) collections. To validate T-DNA insertions, PCR reactions were performed, using the GABI-Kat left border primer 8409 or the SAIL left border primer LB1-short and gene-specific primers spanning the predicted T-DNA insertion site (Supplementary Table 3) (Ülker et al., 2008). For detailed mapping, the PCR products were sequenced. To confirm gene knockout, RNA was extracted from leaves of 14-day-old plants using the Spectrum Plant Total RNA Kit (Sigma). cDNA synthesis was performed using Superscript II reverse transcriptase (Life technologies) according to manufacturer’s instructions. RT-PCR was done with gene-specific primers spanning the T-DNA insertion site. *ACT2* served as a housekeeping reference gene. A homozygous T-DNA knockout line for *PARP3*, *parp3-1* (SALK_108092) has been genotypically analyzed previously (Rissel et al., 2014). *parp* double mutant plants were generated by crossing *parp2-1* with *parp1-1* or *parp3-1*, and *parp3-1* with *parp1-1*. The *parp* triple mutant originated from a cross of *parp2-1 parp1-1* with *parp3-1 parp1-1*.

### Quantification of *PARP* Gene Expression by qRT-PCR

*PARP* gene expression was analyzed on leaves and roots of 14-day-old plate-grown plants. RNA extraction and cDNA synthesis were performed as described above. Gene expression was determined by qRT-PCR as described previously (Lange et al., 2014), running a denaturation step at 95°C for 10 min followed by 40 amplification cycles (95°C for 15 s, 60°C for 1 min). *UBQ10* (At4g05320) was used as housekeeping reference gene (Peiter et al., 2007). Primers are listed in Supplementary Table 3.

### Determination of Stomatal Conductance

Plants were grown in 40 g of a mixture of soil substrate (Tonsubstrat ED 73, Einheitserde Werkverband) and vermiculite in the ratio 2:1. To prevent sciarid infection, Biomükk (BioFA, Germany) was added to the mixture. The pots were covered with a nylon mesh to avoid loss of soil and contamination of leaves. After 5 weeks, plants of similar size for all genotypes were selected. Plant culture was performed in a growth room under short-day conditions (10 h light at 21°C, 14 h dark at 18°C, 130 μmol m^-2^ s^-1^, 65% RH). The 10th, 11th, and 12th leaves of the plants were labeled with a thread. Experiments were conducted on 6-week-old plants. Pots were watered to identical weights until the evening before onset of measurements. Subsequently, water was withheld. Stomatal conductance was measured by using a porometer (AP4, Delta-T Devices, Cambridge, UK) at 11 am for the next 8–10 days. Experiments were performed in triplicate.

### Root Growth Assays

To measure root elongation, surface-sterilized seeds were sown onto 1/2 Murashige and Skoog (MS) agar plates (pH 5.8). Seeds were stratified for 2 days at 4°C. Then agar plates were placed near-vertically into a plant growth cabinet (AR-75, Percival Scientific, Perry, IA, USA) set to long-day conditions (16 h light at 22°C, 8 h dark at 18°C, 130 μmol m^-2^ s^-1^, 65% RH). After 5 days of pre-culture, seedlings were transferred to 1/2 MS agar plates containing the indicated treatment. Root tip position was marked with a felt pen on the plate, and main root length was measured every 2–3 days. After 13–15 days plants were harvested and shoot fresh weight was determined.

### Visualization of Callose Deposition

Callose deposition was determined according to Adams-Phillips et al. (2010). Surface-sterilized seeds were sown onto 1/2 MS agar plates (pH 5.8) containing 2% sucrose. After seed stratification at 4°C for 2 days, plates were placed near-vertically in a plant growth cabinet (ATC-26, Conviron, Winnipeg, MB, Canada) set to short-day conditions (10 h light at 22°C, 14 h dark at 18°C, 130 μmol m^-2^ s^-1^, 65% RH), and plants were grown for 5 days. Thereafter, plants were transferred to liquid 1/2 MS medium containing 1.5% sucrose in 24-well microtiter plates and grown for another 24 h under the same conditions. Subsequently, 1 μM flg22 was added to the liquid medium, and plants were incubated for another 24 h. PARP inhibitors in DMSO or DMSO only were added at indicated time points and concentrations. For fixation, plants were transferred to FAA (formaldehyde, acetic acid, alcohol) solution and incubated for 24 h. Fixed seedlings were stored in 100% ethanol. Before staining, plants were washed in 50% ethanol and 67 mM KH_2_PO_4_ (pH 12). Subsequently, plants were stained in 0.01% aniline blue [in 67 mM KH_2_PO_4_ (pH 12)] for 1 h in the dark. To visualize callose deposition, plants were mounted onto slides in 70% glycerol and 30% staining solution. Six to twelve cotyledons per treatment were visualized under a fluorescence microscope (Axioskop, Carl Zeiss, Jena, Germany) equipped with a UV filter set (No. 9, Zeiss) and photographed with a digital camera (Axiocam MRc, Zeiss) driven by the AxioVision 4.7 software (Zeiss).

### Determination of Poly(ADP-ribosyl)ation

Seeds were sown as a lawn onto the soil substrate-vermiculite mixture described above. After stratification, plants were cultured in a plant growth cabinet (AR-75, Percival Scientific) under long-day conditions (16 h light at 22°C, 8 h dark at 18°C, 130 μmol m^-2^ s^-1^, 65% RH) for 32 days. Then, control plant leaves were cut and frozen in liquid nitrogen. To induce DNA damage and stimulate poly(ADP-ribosyl)ation, plants were treated with 1000 J m^-2^ UV-C light (254 nm) using a UV crosslinker (HL-2000, HybriLinker System, UVP, USA). Leaves were harvested 2 h after UV treatment and frozen in liquid nitrogen. After grinding in liquid nitrogen, nuclear protein was extracted as described by Xia et al. (1997). In brief, 2 g of frozen ground material was homogenized in 4 mL Honda buffer [2.5% Ficoll 400, 5% dextran T40, 400 mM sucrose, 25 mM Tris-HCl pH 7.4, 10 mM MgCl_2_, 10 mM β-mercaptoethanol, protease inhibitor cocktail (P9599, Sigma–Aldrich)]. The homogenate was filtrated through a 70 μm (pore size) nylon net by centrifuging at 30 × *g* and 4°C. The tube was washed with 2 mL Honda buffer. Triton X-100 was added to a final concentration of 0.5%, and samples were incubated on ice for 15 min. Afterward, samples were centrifuged for 5 min at 1500 × *g* and 4°C, and the pellet was washed with Honda buffer containing 0.1 % Triton X-100. The pellet was resuspended in Honda buffer and centrifuged for 5 min at 100 × *g* and 4°C to pellet starch and cellular debris. The nuclei in the supernatant were centrifuged for 10 min at 1800 × *g* and 4°C. The pellet was resuspended in 150 μl Honda buffer. Subsequently, 3 μg protein sample were spotted in triplicate onto a nitrocellulose membrane using a dot blot 96 apparatus (Biometra, Göttingen, Germany) according to manufacturer’s instructions. Equal protein loading was confirmed by Ponceau staining (0.2% Ponceau S in 0.5% acetic acid). Staining was fixed in 0.5% acetic acid. The membrane was washed in PBS and blocked with BSA (Carl Roth). Poly(ADP-ribosyl)ation was visualized using a monoclonal poly(ADP-ribose) antibody (10H, Enzo Life Sciences). After addition of a secondary anti-mouse antibody coupled to a horseradish peroxidase, ECL reagent (250 mg L^-1^ luminol, 0.1 M Tris-HCl pH 8.6, 1% DMSO, 1 g L^-1^ para-coumaric acid) was added. Luminescence was detected and quantified using a photon-counting camera (HRPCS218, Photek, St. Leonards on Sea, UK). The experiment was performed twice with similar results.

### Molecular Modeling of RCD1 and SRO1

The PARP domains of Arabidopsis SRO1 (At2g35510, residues 245–463, according to NCBI) and RCD1 (At1g32230, residues 248–469, according to NCBI) were modeled using the catalytic domains of HsPARP10 [PDB entry 3HKV, Karlberg et al. (unpublished)], GgPARP1 [PDB entry 2PAX, Ruf et al. (1998)], or HsPARP14 [PDB entry 3SE2, Wahlberg et al. (2012)] as template structures. The templates were selected according to their co-crystallized inhibitors 3-aminobenzamide (3-AB), 4-amino-1,8-naphthalimide (4-ANI), and 6-(5H)-phenanthridinone (PHE), respectively. Using YASARA software [YASARA Structure, version 12.11.25, Krieger et al. (2002)], the three-dimensional structures of AtRCD1 and AtSRO1 were built. Since in YASARA template inhibitors are automatically transferred onto the target structure, each homology model includes the corresponding template inhibitor in the target active site. The models were finally refined by the YASARA module md-refinement which performs 20 steps of simulated annealing molecular dynamics simulations.

### Statistical Analysis

In **Figures 4**, **5**, **7**, and **8** and Supplementary Figures 3–5, comparisons of two sample means were performed with two-sided two-sample Welch *t*-tests (Welch, 1947). In **Figures 6** and **11**, one-sided two-sample Welch *t*-tests were performed because an increase in gene expression and photon counts, respectively, was presumed. To compare more than two sample means (**Figure 3**; Supplementary Figure 2), one-factorial analysis of variance was performed at significance level of α = 0.05, followed by a *post hoc* Tukey HSD test (Tukey, 1949), if significant differences were detected. In all figures, an asterisk indicates that the sample mean of the mutant line is significantly different from the sample mean of the wild type for the same treatment and time point (*P* < 0.05). Statistical analysis was performed in R software (version 3.3.2; R Core Team, 2016). Experiments were repeated two to three times with similar results.

## Results

### Expression of *PARP* Genes Is Mostly Unresponsive to Drought, Osmotic, and Salt Stress

**FIGURE 1:**
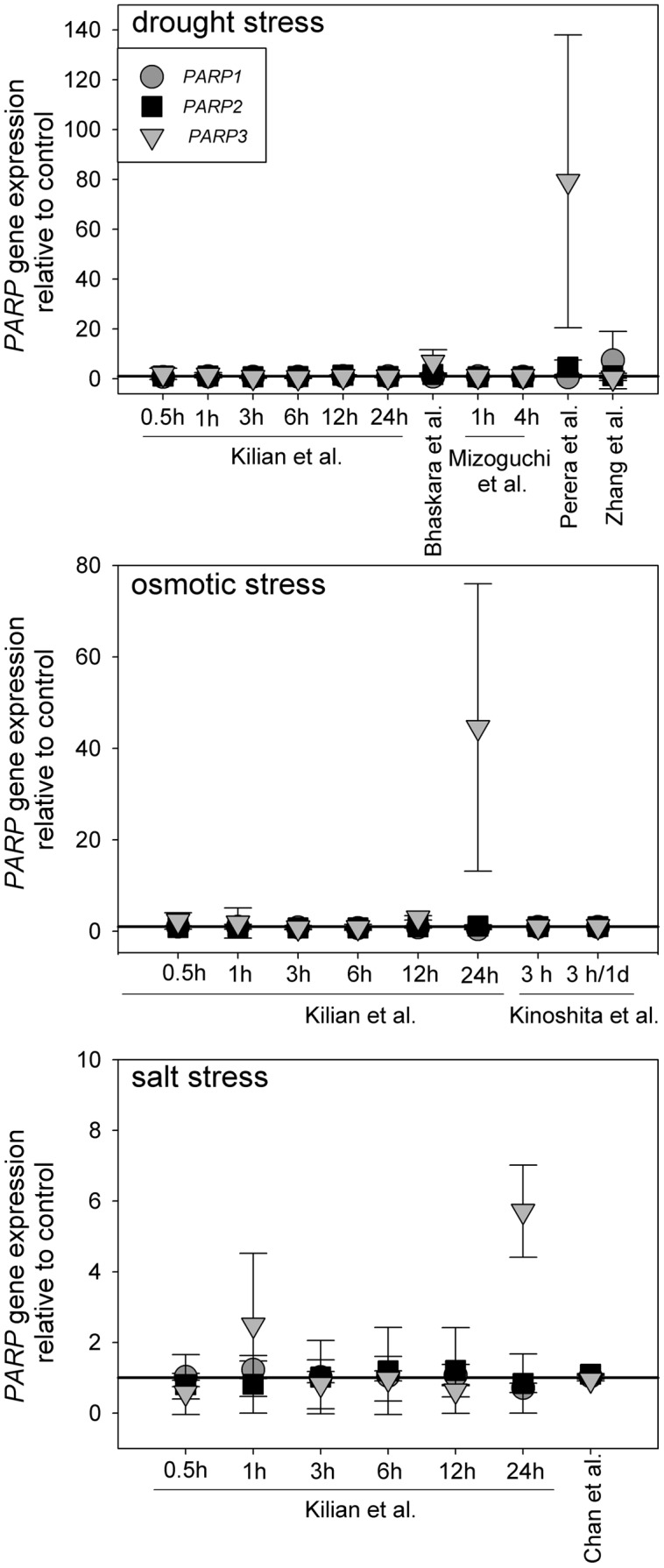
**Publicly available microarray data show a lack of stress-responsiveness of *PARP1* (circles), *PARP2* (squares), and *PARP3* (triangles)**. Data were retrieved from drought, osmotic, and salt stress studies (Kilian et al., 2007; Perera et al., 2008; Zhang et al., 2008; Mizoguchi et al., 2010; Chan et al., 2011; Bhaskara et al., 2012; Kinoshita et al., 2012). Expression levels are relative to control. Error bars represent standard deviation.

Canonical *PARP* genes have been suggested to act as regulators of abiotic stress resistance. Such a role is likely to be reflected in a transcriptional regulation under those conditions. To test this notion, we analyzed a number of microarray experiments in which plants were subjected to drought, osmotic, or salt stress (Kilian et al., 2007; Perera et al., 2008; Zhang et al., 2008; Mizoguchi et al., 2010; Chan et al., 2011; Bhaskara et al., 2012; Kinoshita et al., 2012). The experimental lay-outs and stress intensities varied substantially between those studies. Nevertheless, *PARP1* and *PARP2* were not notably up or downregulated in any of those experiments (**Figure 1**). *PARP3* gave a similar picture in most cases, albeit the upregulation was more pronounced in few instances, reaching up to 80-fold in one drought stress study. However, *PARP3* expression is nearly undetectable under control conditions (Rissel et al., 2014), so that its expression level is very low even under inducing conditions. This general unresponsiveness of *PARP* gene expression to abiotic stress was surprising, considering their presumed involvement in stress responses.

### Identification of T-DNA Insertional Knockout Mutants for *PARP1* and *PARP2* Genes

**FIGURE 2:**
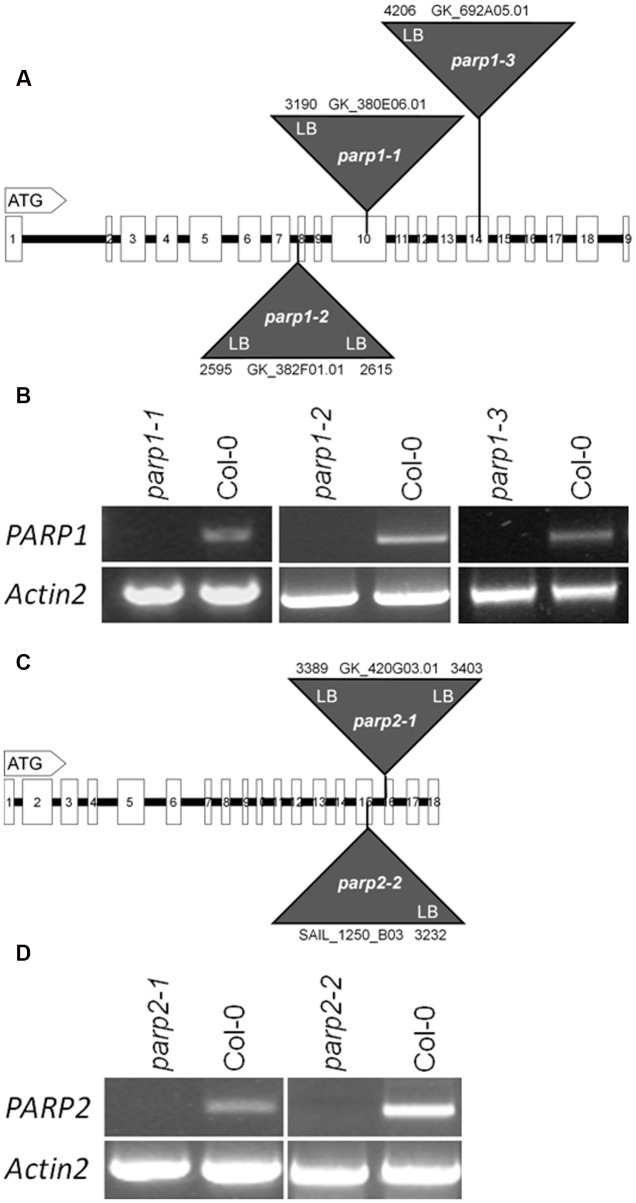
**T-DNA insertion lines were identified for *Arabidopsis thaliana PARP1* and *PARP2*. (A,C)** Model of the genomic regions and the T-DNA insertions in *PARP1* **(A)** and *PARP2* **(C)**. Coding regions are presented by white boxes; introns are shown by a line. Triangles indicate the sites of T-DNA insertions. The numbers indicate the last nucleotide before and the first nucleotide after the insertion, counting from the start condon. LB and RB indicate the left and right border of the T-DNA, as determined by sequencing. **(B,D)** RT-PCR analysis of leaf RNA showing the absence of full-length transcript of the respective *PARP* gene in the mutant lines. *Actin2* served as a control.

To elucidate the involvement of PARP proteins in plant stress responses we searched publicly available T-DNA mutant collections for mutant lines for *PARP1* and *PARP2*. A mutant line for *PARP3* was identified previously (Rissel et al., 2014). In total, seven *parp1* mutant lines were identified carrying T-DNA insertions either in the promoter region of the gene or in its exons (**Figure 2A**; Supplementary Figure 1A). For *PARP2*, five mutant lines were identified with T-DNA insertions showing intron or exon localization (**Figure 2C**; Supplementary Figure 1B). The exact location of T-DNA borders, as determined by sequencing of genomic DNA, can be found in **Figure 2** and Supplementary Figure 1. Since an exon-localized T-DNA insertion is most promising to prevent full-length gene transcription, mutant lines carrying such an insertion were further characterized. The *PARP1* gene consists of 19 exons (**Figure 2A**). The T-DNA insertions in the *parp1-1*, *parp1-2*, and *parp1-3* mutants are located in exon 10, 8, and 14, respectively. The *PARP2* gene consists of 18 exons (**Figure 2C**). The T-DNA insertions in the *parp2-1* and *parp2-2* mutants are located in exon 16 and 15, respectively. Semi-quantitative RT-PCR analysis on leaves of 2-week-old plants confirmed the lack of *PARP1* and *PARP2* transcripts in those *parp1* and *parp2* T-DNA lines, respectively (**Figures 2B,D**).

### A Unified Nomenclature for Arabidopsis *PARP* Genes and *parp* Mutants

Some of the T-DNA lines shown in **Figure 2** and Supplementary Figure 1 have been employed in previous analyses, but their nomenclature has been redundant and inconsistent so far. In combination with the above-mentioned inconsistency of the gene nomenclature (Supplementary Table 1), this complicates the integration and discussion of published experimental data. We have therefore compiled all publications involving *parp* mutants and suggest a unified mutant nomenclature, which is shown in Supplementary Table 2. This nomenclature is consistent with the annotation in the TAIR^1^ and Araport^2^ databases.

### Mutation of Individual *PARP* Genes Does Not Alter Performance of Plants Exposed to Various Abiotic Stresses

**FIGURE 3:**
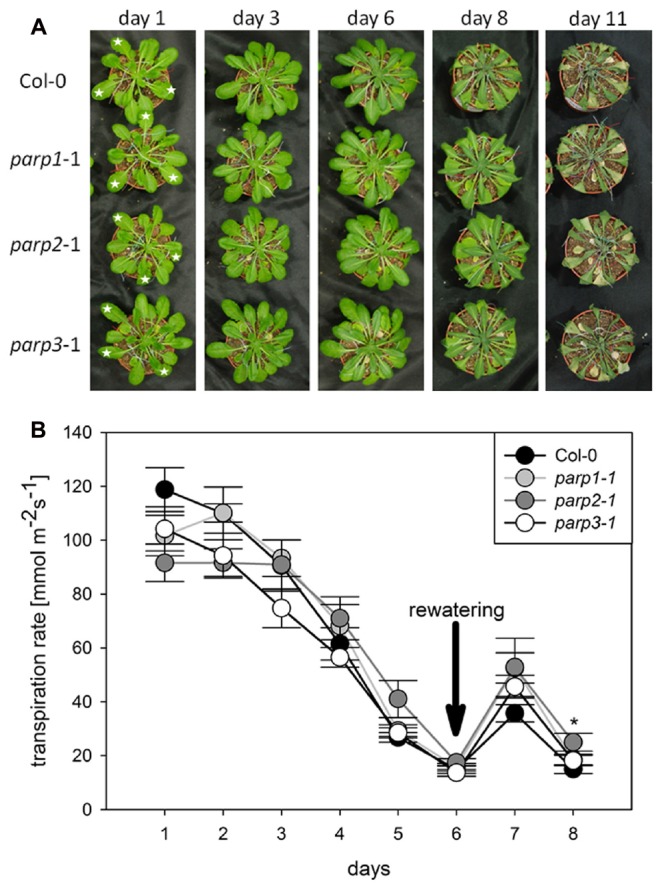
**Desiccation tolerance and stomatal conductance are not altered in *parp* mutant plants compared to the wild type. (A)** Images of soil-grown Col-0 wild type and *parp* mutant plants at different stages of desiccation. **(B)** Transpiration during desiccation determined by porometry. Three leaves of a similar developmental stage were measured [marked by stars in **(A)**]. After 6 days plants were re-watered with 20 mL water. Data represent the sample means ± SE of 3–4 plants per line and three leaves per plant. Data for individual leaves are shown in Supplementary Figure 2.

To analyze the link between PARPs and drought responses, we performed a soil desiccation experiment comparing 6-week-old wild type (Col-0) and *parp1-1*, *parp2-1*, and *parp3-1* mutant plants. Surprisingly, all three *parp* mutant lines did not show a visibly enhanced tolerance to this stress as compared to the Col-0 plants (**Figure 3A**). Stomatal conductance of the 10th, 11th, and 12th leaf was measured during the desiccation period using porometry (Supplementary Figure 2). Since transpirational water loss for the three leaves was similar, their mean values were calculated. The three *parp* mutant lines showed a similar transpiration rate as the wild type (**Figure 3B**).

**FIGURE 4:**
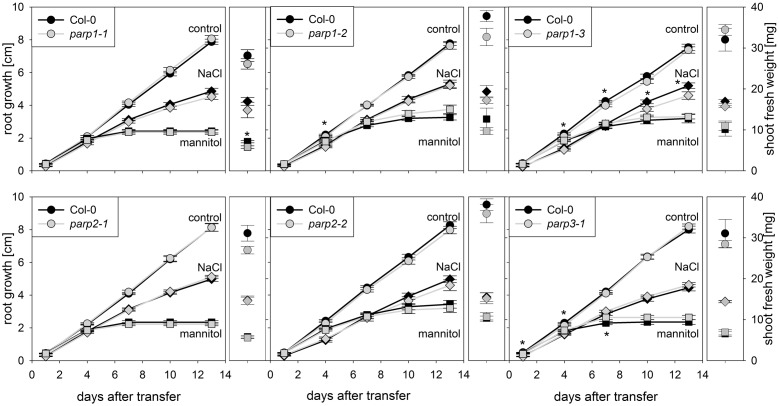
**Root growth of *parp* mutant seedlings subjected to salt or osmotic stress is not altered compared to the wild type**. Root growth (large panels) of Col-0 and *parp* mutants on control plates (circles), and plates supplemented with 100 mM NaCl (diamonds) or 100 mM mannitol (squares). Shoot fresh weight was determined at the end of the experiment (small panels). Data represent the sample means ± SE of 15 plants per line.

Drought and osmotic stress affect not only shoot growth and transpiration, but also primary root elongation. To monitor root and shoot growth in response to abiotic stresses, *parp* mutants and wild type plants were grown on agar plates. To mimic drought stress, mannitol was applied as osmoticum. Furthermore, NaCl and H_2_O_2_ were applied as abiotic stress factors. Under control conditions, all plant genotypes showed similar root growth rates (**Figure 4**; Supplementary Figure 3). Mannitol (100 mM), NaCl (100 mM), and H_2_O_2_ (0.5 mM) treatments reduced root growth and shoot fresh weight. Unexpectedly, *parp* mutants did not show any pronounced and consistent differences to the Col-0 plants in root growth and shoot weight in response to the applied stress treatments.

**FIGURE 5:**
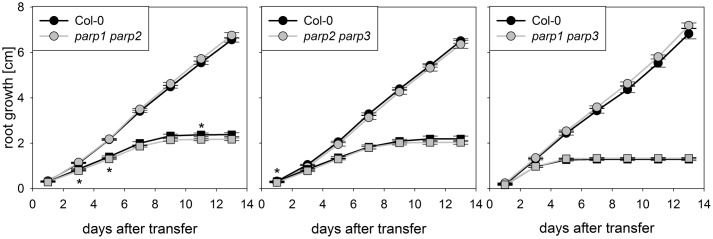
**Root growth of *parp* double mutant seedlings subjected to osmotic stress is not altered compared to the wild type**. Root growth of Col-0 and *parp* double mutants on control treatment (circles) or 100 mM mannitol (squares). Data represent the sample means ± SE of 15 plants per line.

The *parp* mutant plants did not display the hypertolerance to abiotic stress that we expected from previous studies which mostly employed PARP inhibitors and knockdown lines. A possible reason for this might be a functional redundancy of the three PARP proteins. To further elucidate this, *parp1-1*, *parp2-1*, and *parp3-1* mutants were crossed with each other to generate double mutant lines, which were subjected to an osmotic stress assay. On agar plates containing 100 mM mannitol, root growth and shoot fresh weight of the double mutants was not different from that of the wild type (**Figure 5**).

### *parp* Triple Knockout Does Not Alter Plant Response to Various Abiotic Stresses

**FIGURE 6:**
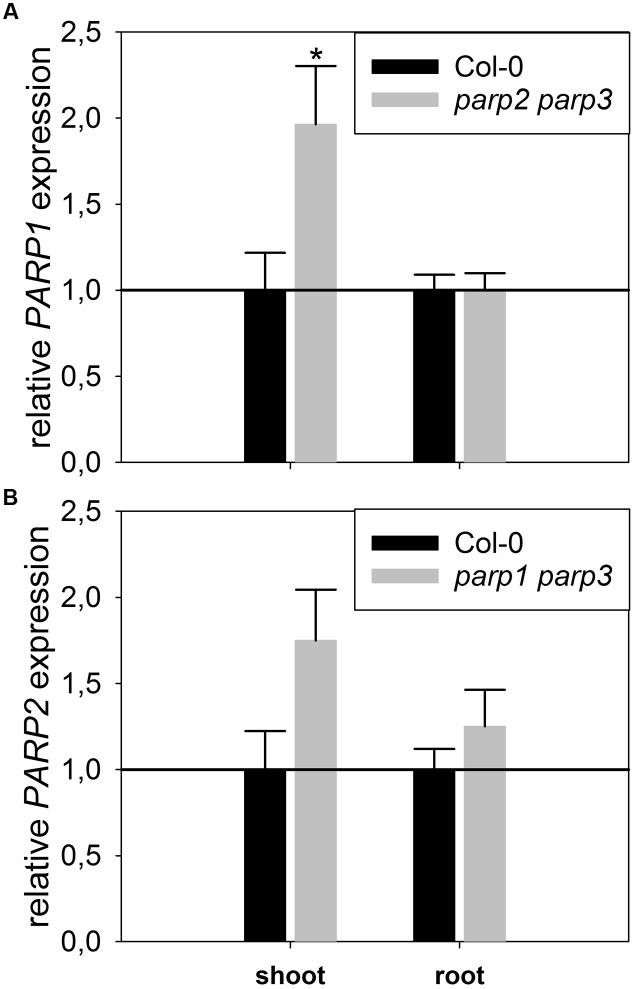
**Expression of *PARP1* and *PARP2* is induced in *parp* double mutants lacking the other two *PARP* genes**. A qRT-PCR was performed on shoots of plate-grown seedlings of *parp2-1 parp3-1* **(A)** and *parp1-1 parp3-1* **(B)** using *AtUBI10* as reference gene. *PARP3* gene expression was below detection limit. Data represent the sample means ± SE of four plants per line.

**FIGURE 7:**
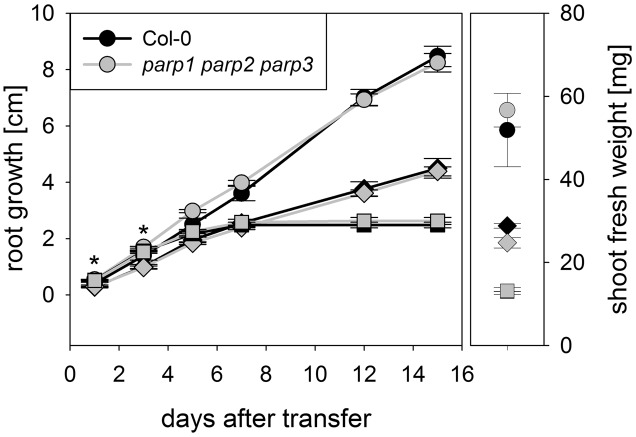
**Root growth of *parp1-1 parp2-1 parp3-1* mutant plants exposed to salt or osmotic stress is not altered compared to the wild type**. Root growth (large panel) of Col-0 and *parp1-1 parp2-1 parp3-1* mutants on control plates and on plates supplemented with 100 mM NaCl (diamonds) or 100 mM mannitol (squares). Shoot fresh weight was determined at the end of the experiment (small panel). Data represent the sample means ± SE of 15 plants per line.

To determine whether expression of the residual third *PARP* gene may be upregulated in the double mutants, its transcript level was determined in 2-week-old plants. In *parp2-1 parp3-1* mutant plants, *PARP1* expression was doubled in shoots, while the expression in roots was similar between wild type and double mutant (**Figure 6**). The *parp1-1 parp3-1* double mutation also led to a tendentially increased expression of *PARP2* in shoots (1.7 fold; *P* = 0.059). *PARP3* expression was found to be below the detection level in leaves of the *parp1-1 parp2-1* mutant, as it was in the wild type. These data indicate that, at least in the double mutants involving *parp3*, the residual *PARP* gene may at least partially compensate for the knocked-out ones. In addition, since PARPs have been described to be post-translationally activated upon stress, it could not be fully excluded that PARP3 activity is induced in the *parp1-1 parp2-1* double mutants (Bürkle and Virag, 2013). Therefore, *parp1-1 parp2-1* and *parp1-1 parp3-1* double mutant plants were crossed to generate a *parp* triple mutant. This line was subjected to osmotic, salt, and oxidative stress assays as described above. Like the *parp* single and double mutant plants, *parp1-1 parp2-1 parp3-1* plants did not show an enhanced performance compared to the wild type under any of those conditions (**Figure 7**; Supplementary Figure 4).

**FIGURE 8:**
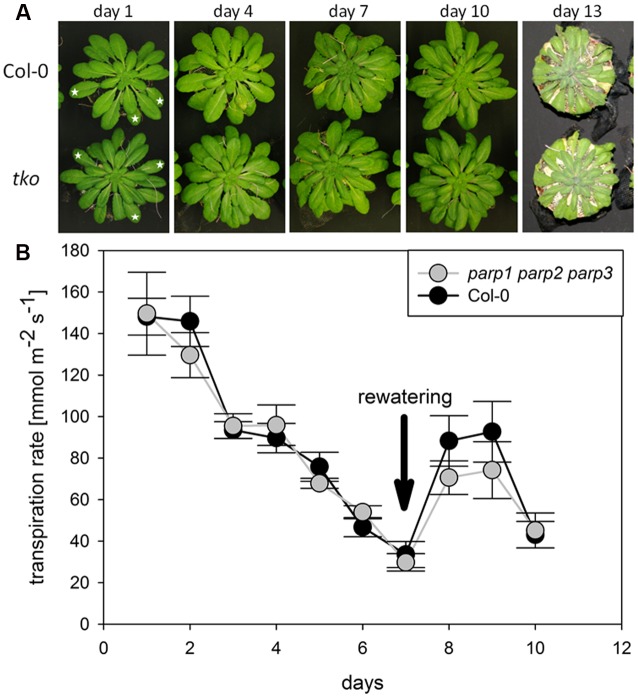
**Desiccation tolerance and stomatal conductance are not altered in *parp1-1 parp2-1 parp3-1* mutant plants compared to the wild type. (A)** Images of soil-grown Col-0 wild type and *parp1-1 parp2-1 parp3-1* mutant plants at different stages of desiccation. **(B)** Transpiration during desiccation determined by porometry. Three leaves of a similar developmental stage were measured [marked by stars in **(A)**]. After 7 days plants were re-watered with 20 mL water. Data represent the sample means ± SE of three plants per line and three leaves per plant. Data for individual leaves are shown in Supplementary Figure 5.

We analyzed if the lack of all three *PARP* genes had an impact on the response of soil-grown adult Arabidopsis plants to drought stress. Triple mutants were subjected to desiccation as described above for the single mutant plants, and plant phenotype and transpiration were monitored. As before, triple mutants did not show a visibly enhanced stress tolerance (**Figure 8A**). Also, both genotypes showed similar transpiration rates (**Figure 8B**; Supplementary Figure 5). Hence, it could not be confirmed in any of our experiments that abiotic stress tolerance is improved by an absence of functional *PARP* genes.

### Pharmacological PARP Inhibition but Not Genetic Knockout Blocks flg22-Induced Callose Deposition

**FIGURE 9:**
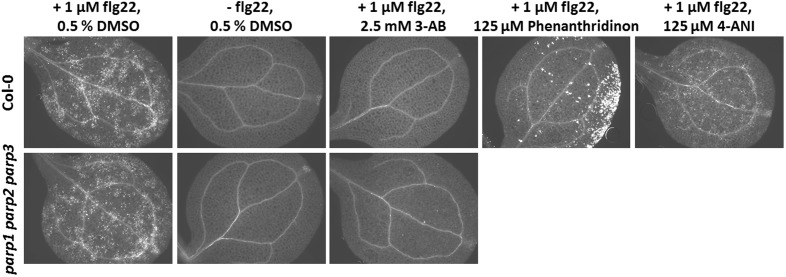
**Pharmacological PARP inhibition but not *PARP* gene knockout blocks flg22-induced callose deposition**. Callose deposition in 7-day-old Col-0 or *parp1-1 parp2-1 parp3-1* mutant seedlings was determined 24 h after the indicated treatment. Images are representative of a minimum of six cotyledons per treatment and genotype.

Apart from abiotic stress, PARP action has been linked to biotic stress responses. Previously, PARP inhibition by the PARP inhibitor 3-AB was shown to block flg22-induced, but not wounding-induced, callose deposition in cotyledons of Col-0 seedlings (Adams-Phillips et al., 2010). Thus, PARP proteins seem to specifically interact with the flg22-triggered defense pathway. To confirm this, we first tested other known PARP inhibitors for their potential to block flg22-induced callose deposition. Similar to 3-AB, 6-(5H)-phenanthridinone blocked the callose deposition in Col-0 cotyledons (**Figure 9**). Very bright fluorescent spots which appeared on the edges of the cotyledons after phenanthridinone treatment were due to precipitation of the inhibitor. Interestingly, 4-ANI, another PARP inhibitor, did not prevent callose deposition in response to flg22 treatment (**Figure 9**). In summary, two different known PARP inhibitors were effective in blocking callose deposition, which may indeed point to a role of PARPs in plant response to bacterial attack. A similar effect was therefore expected for the *parp* triple mutant. Surprisingly, the pattern of callose deposition was not altered in cotyledons of this line, as compared to the wild type (**Figure 9**). The application of 3-AB to flg22-treated *parp* triple mutant seedlings evoked the expected blocking of callose deposition. These data indicate that the employed inhibitors act on targets other than or in addition to classical PARPs, affirming a similar assumption based on previous studies with PARP inhibitors (Geissler and Wessjohann, 2011).

### PARP Inhibitors Are Likely to Interact with Other Plant Proteins

**FIGURE 10:**
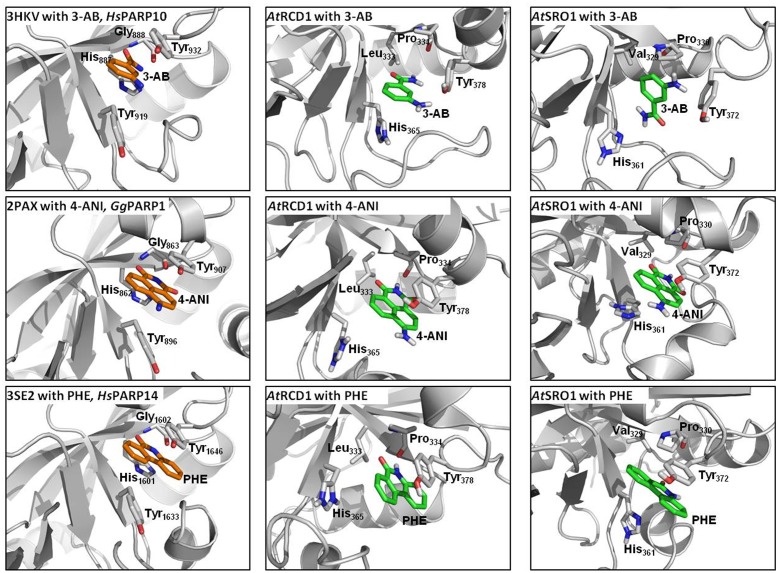
**Comparisons of active sites and inhibitor binding in crystal structures of animal ADP ribosyltransferase proteins, AtRCD1, and AtSRO1**. Left panels show crystal structures of HsPARP10, GgPARP1, and HsPARP14 with co-crystallized inhibitors 3-aminobenzamide (3-AB), 4-amino-1,8-naphthalimide (4-ANI), and 6-(5H)-phenanthridinone (PHE), respectively. Center and right panels show homology models of AtRCD1 and AtSRO1 with bound inhibitors. Despite different amino acid motifs, the inhibitors can be bound in the same manner as in the template structures.

Proteins of the RCD1/SRO family contain a presumed catalytic PARP domain but not the regulatory one [Jaspers et al. (2010b); Supplementary Figure 6]. To analyze if pharmacological PARP inhibitors, commonly employed to infer roles of PARPs in plants, potentially interact with these proteins, the PARP domains of RCD1 and SRO1 were modeled, and their active sites were analyzed with respect to the ability to bind 3-AB, 4-ANI, and 6-(5H)-phenanthridinone (**Figure 10**). The structures of the six homology models can be inspected in detail on the pdb files included in the Supplementary Material. Despite low overall sequence identities between the templates and RCD1 or SRO1 (between 15.8 and 21.6%, depending on target and alignment), active site inspections confirmed that all three inhibitors could be bound via the same type of interactions that are observed in X-ray structures of ADP ribosyltransferase-type PARPs, e.g., HsPARP10 (including 3-AB), GgPARP1 (including 4-ANI), and HsPARP14 (including 6-(5H)-phenanthridinone). In HsPARP10 or GgPARP1, the nicotinamide moiety of inhibitors is recognized by two hydrogen bonds of a glycine residue. Further stabilization is mediated through stacking between hydrophobic tyrosine side chains. In RCD1 and SRO1, despite a three-dimensional conservation of the active site, both polar and non-polar interaction patterns are disrupted by exchanges in primary sequence. In RCD1 and SRO1, the conserved glycine of animal PARPs is exchanged by a proline (Pro_334_ and Pro_330_, respectively). This results in only one possible hydrogen bond between RCD1 or SRO1 and the inhibitor (mediated by the proline backbone oxygen atom). Alternatively, after performing the md-refinement simulations, 3-AB adopts a pose in AtRCD1 with preferred hydrophobic interactions between proline and the phenyl moiety of 3-AB. The only amino acid in the classical PARP motif (Ferraris, 2010) that is conserved in RCD1 and SRO1 is a tyrosine (Tyr_378_ and Tyr_372_, respectively), suggested to be responsible for π–π interactions between the inhibitor and the receptor. The same interaction pattern resulted also for the binding pose of 4-ANI and phenanthridinone. Another tyrosine which is conserved in animal PARPs is replaced by a histidine in plant RCD1 or SRO1 (His_365_ and His_361_, respectively), which still allows the inhibitors to be stacked between two residues in the same way as it is in animal PARPs. Furthermore, in animal PARPs there is a conserved histidine (e.g., His_862_ in HsPARP1) in close proximity to the binding site of the inhibitors which is necessary for specific activity (Marsischky et al., 1995). This amino acid is replaced by a Leu_333_ in AtRCD1 and Val_329_ in AtSRO1. However, these replacements do not influence the putative binding of the inhibitors. In all the models the binding site is accessible for the inhibitors to penetrate.

In summary, although we do not exclude that some slightly different docking poses of the ligands in the binding site may occur, it could be shown that in principle all these ligands may act as inhibitors for AtSRO1 and AtRCD1 as well. The possibility to bind the inhibitors does not necessarily imply that the proteins have an activity as PARP enzymes. Even if RCD1 and SRO1 would act merely as non-enzymatic scaffolding proteins, the binding of an inhibitor may disturb protein-protein interactions and, hence, protein function.

### PARP Activity Is Constitutively Upregulated in a *parp* Triple Knockout Mutant

**FIGURE 11:**
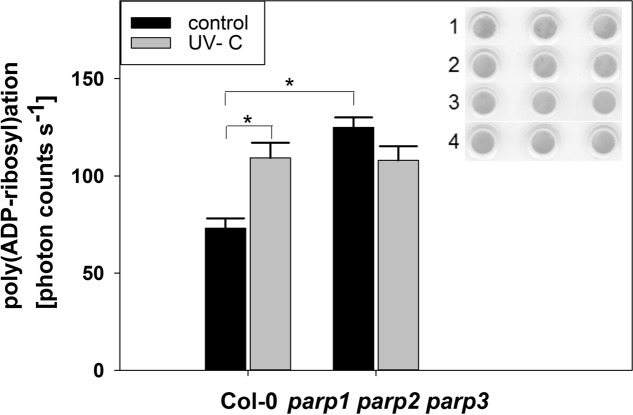
**Poly(ADP-ribosyl)ation is enhanced by UV radiation and in *parp* triple knockout plants**. PARylation of nuclear proteins was analyzed in 32-day-old leaf material using a poly(ADP-ribose) monoclonal antibody. The insert shows Ponceau staining of protein loaded onto the nitrocellulose membrane (1: Col-0 control, 2: *parp1-1 parp2-1 parp3-1* control, 3: Col-0 UV-treated, 4: *parp1-1 parp2-1 parp3-1* UV-treated). Error bars represent standard error (*N* = 3).

The application of PARP inhibitors has been frequently demonstrated to modulate plant responses to biotic and abiotic cues. This differs from our findings on *parp* mutants, and our modeling analysis indicated that inhibitors may also target non-PARP proteins. Because inhibitor effects are still likely to be caused by an interference with enzymatic activity, e.g., a reduction in protein poly(ADP-ribosyl)ation, we tested whether this activity is completely abolished by genetic deletion of all three classical *PARP*s. To this end, we performed a dot-blot assay employing a monoclonal poly(ADP-ribose) antibody (**Figure 11**). Since equal protein concentrations were spotted onto the nitrocellulose membrane and samples were processed identically, the background signal from the antibody is expected to be similar in all samples. In wild type plants, protein poly(ADP-ribosyl)ation was induced by UV light stress, which is expected from its role in DNA damage repair. Most surprisingly, under unstressed conditions, poly(ADP-ribosyl)ation was not found to be abolished, but to be even increased in the triple *parp* mutant as compared to the wild type. This activity was not further stimulated by UV illumination. This result further supports the presence of additional proteins with PARP activity in Arabidopsis, whose activity is increased by the knockout of the classical *PARP* genes.

## Discussion

### *PARP* Genes Do Not Play a Universal Role in Growth under Abiotic Stress Conditions

Under the conditions that we tested in this study, *parp* T-DNA insertion mutants did not exhibit altered stress responses compared to wild type plants (**Figures 3** and **4**). This was also the case in all double mutant combinations (**Figure 5**) and in a triple mutant (**Figures 7** and **8**) and was therefore not due to functional redundancy, although our expression analysis pointed to some degree of transcriptional feedback (**Figure 6**). These findings apparently disagree with previous studies employing plants with genetically downregulated PARP activity, from which a negative role of this gene family in abiotic stress resistance was inferred (De Block et al., 2005; Jansen et al., 2009; Schulz et al., 2012). This discrepancy may be explained by different experimental conditions and/or plant genotypes and indicates that the role of PARPs as factors of growth and stress responses is less universal than commonly assumed. For example, De Block et al. (2005) worked with lines derived from the *Arabidopsis thaliana* C24 ecotype in their desiccation experiments, whereas in the present study *A. thaliana* mutants in the Col-0 background were used. General differences in stress tolerance between both genotypes are not unlikely, since the C24 ecotype has been described to be more susceptible to cold stress and UV-B irradiation as compared to Col-0 (Klotke et al., 2004; Kalbina and Strid, 2006). Apart from that, different methodological approaches to alter *PARP* gene expression were employed. In the present study, we analyzed T-DNA insertion mutants, while plants carrying hairpin constructs have been used in other studies (De Block et al., 2005). Expression of the target gene is fully blocked in insertional T-DNA knockout mutants, whereas RNAi-mediating hairpin constructs lead to a partial knockdown and insert randomly into the plant genome, which may potentially affect other genes. Hence, ecotype and genetic modification may explain some of the discrepancies between previous reports and the results we obtained. In addition, growth conditions and age varied between the different studies showing an effect or no effect of *PARP* interference. However, we employed two very different systems, growing the plants on agar plates and on soil, without detecting a role of this gene family. In conclusion, enhanced stress response by a repressed *PARP* expression appears to be a conditional phenotype. This notion is supported by a general unresponsiveness of *PARP* gene expression to osmotic, drought, or salt stress (**Figure 1**).

### Pharmacological PARP Inhibitors May Have Off-Target Effects

Apart from the genetic interference with *PARP* genes, pharmacological inhibition has been used in the past to elucidate the role of plant PARP proteins in stress responses. In those studies, PARP inhibitors known to be potent in human cells were employed. Positive effects of pharmacological PARP inhibition on plant performance under stress have been described for several plant species, various developmental stages, and different stress factors, such as oxidative stress, osmotic stress, or salt stress (De Block et al., 2005; Geissler and Wessjohann, 2011; Schulz et al., 2012). Conversely, pharmacological PARP inhibition negatively interfered with plant immune responses to pathogen-associated molecular patterns, such as flg22 or elf18 (Adams-Phillips et al., 2010). This was confirmed in the present study; two PARP inhibitors blocked flg22-induced callose deposition (**Figure 9**). However, the genetic abolition of all three *PARP* genes did not provoke this effect. These findings indicate that pharmacological PARP inhibition is more effective than genetic reduction of PARP activity, which points to the existence of other or additional proteins targeted by pharmacological PARP inhibitors. This is in agreement with a previous study of PARP inhibitor action on plants by some of us, which casted a first doubt on PARP inhibition as a cause for drought stress tolerance (Geissler and Wessjohann, 2011). In the current study, this notion is supported by both, experimental evidence and computer modeling. Experimentally, we made the surprising observation that PARP activity is not abolished, but instead constitutively induced in a *parp* triple knockout line (**Figure 11**). Hence, there are bound to be further proteins with PARP activity that may be targeted by the employed inhibitors. Possible candidates are members of the SRO protein family, which have been assigned key roles in stress responses of Arabidopsis, wheat, and rice (Katiyar-Agarwal et al., 2006; Teotia and Lamb, 2009; Liu et al., 2014; You et al., 2014). SRO proteins contain a presumed catalytic PARP domain, albeit *in vitro* assays failed to show any enzymatic activity (Jaspers et al., 2010b). Although overall protein sequence similarities to crystallized PARP proteins were low, homology modeling of the catalytic domain of SRO proteins was possible (**Figure 10**). Those PARP inhibitor modeling studies showed that 3-AB, 4-ANI, and phenanthridinone should be able to bind to the binding pocket of RCD1 and SRO1, the best-characterized members of the SRO protein family with partially redundant functions.

In addition to the catalytic PARP domain, both proteins also contain an N-terminal WWE domain and a C-terminal RST domain (Supplementary Figure 6), which are known to mediate protein-protein interactions. Prominent interaction partners of RCD1 and SRO1 are DREB2-type transcription factors (Jaspers et al., 2009), central regulators of drought, salt, and heat stress responses. DREB2A is regulated by protein stability (Sakuma et al., 2006), and there is substantial evidence that binding of RCD1 to DREB2A designates the protein to degradation (Vainonen et al., 2012). Hence, RCD1 is a negative regulator of DREB2A. A similar role for SRO1 has not yet been investigated, but may be assumed from its interaction with DREB2A and its partial functional redundancy with RCD1 (Jaspers et al., 2009).

The PARP domain has been suggested to facilitate complex formation of SRO proteins with their interaction partners (Jaspers et al., 2010a). This domain would hence be required for the designation of DREB2A to degradation by binding to RCD1 and possibly SRO1. This, in turn, means that occupation of the PARP domain by pharmacological compounds is likely to increase the stability of DREB2A by blocking its interaction with RCD1. Therefore, one potential effect of PARP inhibitors may be an increased DREB2A activity, leading to the commonly observed increased stress resistance. However, in addition to altered stress responses, *rcd1* knockout mutants show severe developmental defects (Fujibe et al., 2004; Jaspers et al., 2009; Teotia and Lamb, 2009; Hiltscher et al., 2014), which are not induced by PARP inhibitors (De Block et al., 2005; Adams-Phillips et al., 2010; Geissler and Wessjohann, 2011; Schulz et al., 2012). This discrepancy may be explained by the fact that, in contrast to a genetic knockout of *RCD1*, the RST domain is still present in the PARP-inhibitor-complexed RCD1. Hence, interactions with transcription factors involved in plant development may still be possible.

## Conclusion

The lack of stress-related phenotypes in *parp* mutants, the higher effectiveness of pharmacological PARP inhibition, and the PARP activity in a *parp* triple knockout mutant indicate that additional proteins are affected by the inhibitors. We identified RCD1 and SRO1 as possible candidates. Further research is required to investigate this likely interaction, which may eventually be harnessed to improve the performance of field crops under stress conditions.

## Author Contributions

DR performed experiments; PH, LW, and WB conducted modeling analyses; DR, PH, KT, WB, LW, and EP designed and oversaw the research and analyzed data; DR and EP wrote the article with contributions of PH, KT, LW, and WB.

## Conflict of Interest Statement

The authors declare that the research was conducted in the absence of any commercial or financial relationships that could be construed as a potential conflict of interest.

## Footnotes

1 http://www.arabidopsis.org

2 http://www.araport.org
