# Supplementary material for: No Silver Bullet – Canonical Poly(ADP-Ribose) Polymerases (PARPs) Are No Universal Factors of Abiotic and Biotic Stress Resistance of Arabidopsis thaliana
Source: Front Plant Sci. 2017 Feb 6;8:59. doi: 10.3389/fpls.2017.00059 (PMC5292411; doi:10.3389/fpls.2017.00059)
Supplement: Supplementary file 2 [file Image_1.pdf]

Supplementary Material

No silver bullet - Canonical Poly(ADP-Ribose) Polymerases (PARPs) are no universal factors of abiotic and biotic stress responses of *Arabidopsis thaliana*

Dagmar Rissel, Peter P. Heym, Kathrin Thor, Wolfgang Brandt, Ludger A. Wessjohann and Edgar Peiter*

*** Correspondence:** Edgar Peiter: edgar.peiter@landw.uni-halle.de

Supplementary Presentations. Zip archive containing pdb files of protein models shown in Figure 10. (AtRCD1_3AB.pdb; AtRCD1_4ANI.pdb; AtRCD1_PHE.pdb; AtSRO1_3AB.pdb; AtSRO1_4ANI.pdb; AtSRO1_PHE.pdb)

Supplementary Table 1. The nomenclature of *Arabidopsis thaliana PARP1* and *PARP2* has been inconsistent in the literature.

**Reference**	**At2g31320**	**At4g02390**
this work	PARP1	PARP2
Zhang et al. (2015)<br>Sci. Rep. 5:15892	PARP1	PARP2
Pham et al. (2015)<br>Plant Mol. Biol., 89: 319-338	PARP2	PARP1
Song et al. (2015)<br>PLOS Genet. 11: e1005200	PARP1	PARP2
Feng et al. (2015)<br>PLOS Genet. 11: e1004936	PARP1	PARP2
Boltz et al. (2014)<br>PLOS ONE 9: e88872	PARP2	PARP1
Jia et al. (2013)<br>Plant Mol. Biol. 82: 339-351	PARP1	PARP2
Schulz et al. (2012)<br>PLOS ONE 7: e37287	PARP2	PARP1
Lamb et al. (2012)<br>Cell Mol. Life Sci. 69: 175-189	PARP2	PARP1
Briggs and Bent (2011)<br>Trends Plant Sci. 16: 372-380	PARP2	PARP1
Pellny et al. (2009)<br>Mol. Plant 2: 442-456	PARP1	PARP2
Ogawa et al. (2009)<br>Plant J. 57: 289-301	PARP1	PARP2
Vanderauwera et al. (2007)<br>PNAS 104: 15150-15155	PARP2	PARP1
De Block et al. (2005)<br>Plant J. 41: 95-106	PARP2	PARP1
Doucet-Chabeaud et al. (2001)<br>Mol. Genet. Genom. 265: 954-963	PARP1	PARP2

Supplementary Table 2. A unified nomenclature of Arabidopsis *parp* mutants.

**PARP1 (At2g31320)**		
**Mutant**	**Collection**	**previously published as**
parp1-1	GABI_380E06	*parp2* [1]; *parp1-1* [7]
parp1-2	GABI_382F01	*parp1-2* [7]
parp1-3	GABI_692A05	*atparp1* [2]; *parp1* [3]; *parp1* [8]
parp1-4	SALK_145153	*parp2* [6]
parp1-5	SALK_111410	*parp-2* [4]
parp1-6	SALK_109413	
parp1-7	SALK_141560	
**PARP2 (At4g02390)**		
**Mutant**	**Collection**	**previously published as**
parp2-1	GABI_420G03	*parp2-1* [7]
parp2-2	SAIL_1250_B03	*parp-3* [4]
parp2-3	SALK_140400	*parp1* [1]; *atparp2* [2]; *parp2* [3]; *parp2* [8]
parp2-4	SALK_097261	*parp1* [6]
parp2-5	SAIL_683_F10	
**PARP3 (At5g22470)**		
**Mutant**	**Collection**	**previously published as**
parp3-1	SALK_108092	*parp3-1* [5]; *parp3* [6]; *parp3* [8]
parp3-2	SAIL_632_D07	*parp-1* [4]

[1] Boltz et al. (2014), PLOS ONE, 9: e88872

[2] Feng et al. (2015), PLOS Genet. 11(1): e1004936

[3] Jia et al. (2013), Plant Mol. Biol. 82: 339-351

[4] Pham et al. (2015), Plant Mol. Biol. 89: 319-338

[5] Rissel et al. (2014), Plant Biol. 16: 1058-1064

[6] Schulz et al. (2012), PLOS ONE 7: e37287

[7] Song et al. (2015), PLOS Genet. 11: e1005200

[8] Zhang et al. (2015), Sci. Rep. 5:15892

Supplementary Table 3. Primers used in this work.

PCR screening and RT-PCR	*parp1-1*_for	ACTCCTCAAGGAGTGAAAGGC
	*parp1-1*_rev	ATCTCGAACTCCATCATTGC
	*parp1-2*_for	TGGAGCAAATGTTCTCATTCC
	*parp1-2*_rev	GATGCTTACAATGTCCAACGG
	*parp1-3*_for	TTGAGGCATTGACGGAGATAC
	*parp1-3*_rev	TTTCTCCCAATGCAACTTCAC
	GABI_8409	ATATTGACCATCATACTCATTGC
gene expression	*PARP1*_rt_for	GAAATACTAAGGAAAGGCAACCAT
	*PARP1*_rt_rev	TGTCAGTCCACAAACAACCAAA
PCR screening and RT-PCR	*parp2-1*_for	AGAACACTCATGCAAAGACGC
	*parp2-1*_rev	ACGCATCTTGATTTGTTCCAC
	*parp2-2*_for	AGAACACTCATGCAAAGACGC
	*parp2-2*_rev	AAGTGGAACAACAACACCGTC
	GABI_8409	ATATTGACCATCATACTCATTGC
	SAIL_LB1_short	CAGAAATGGATAAATAGCCTTGCTTC
	SAIL_LB3_short	GCATCTGAATTTCATAACCAATC
gene expression	*PARP2*_rt_for	GGCAAGATAAGCAAGTCCACA
	*PARP2*_rt_rev	ACTCAGTTCCTCAAGCCTCGT
reference genes	*ACT2*_rt_for	TCCCTCAGCACATTCCAGCAGAT
	*ACT2*_rt_rev	AACGATTCCTGGACCTGCCTCATC
	*UBQ10*_rt_for	CACACTCCACTTGGTCTTGCGT
	*UBQ10*_rt_rev	TGGTCTTTCCGGTGAGAGTCTTCA

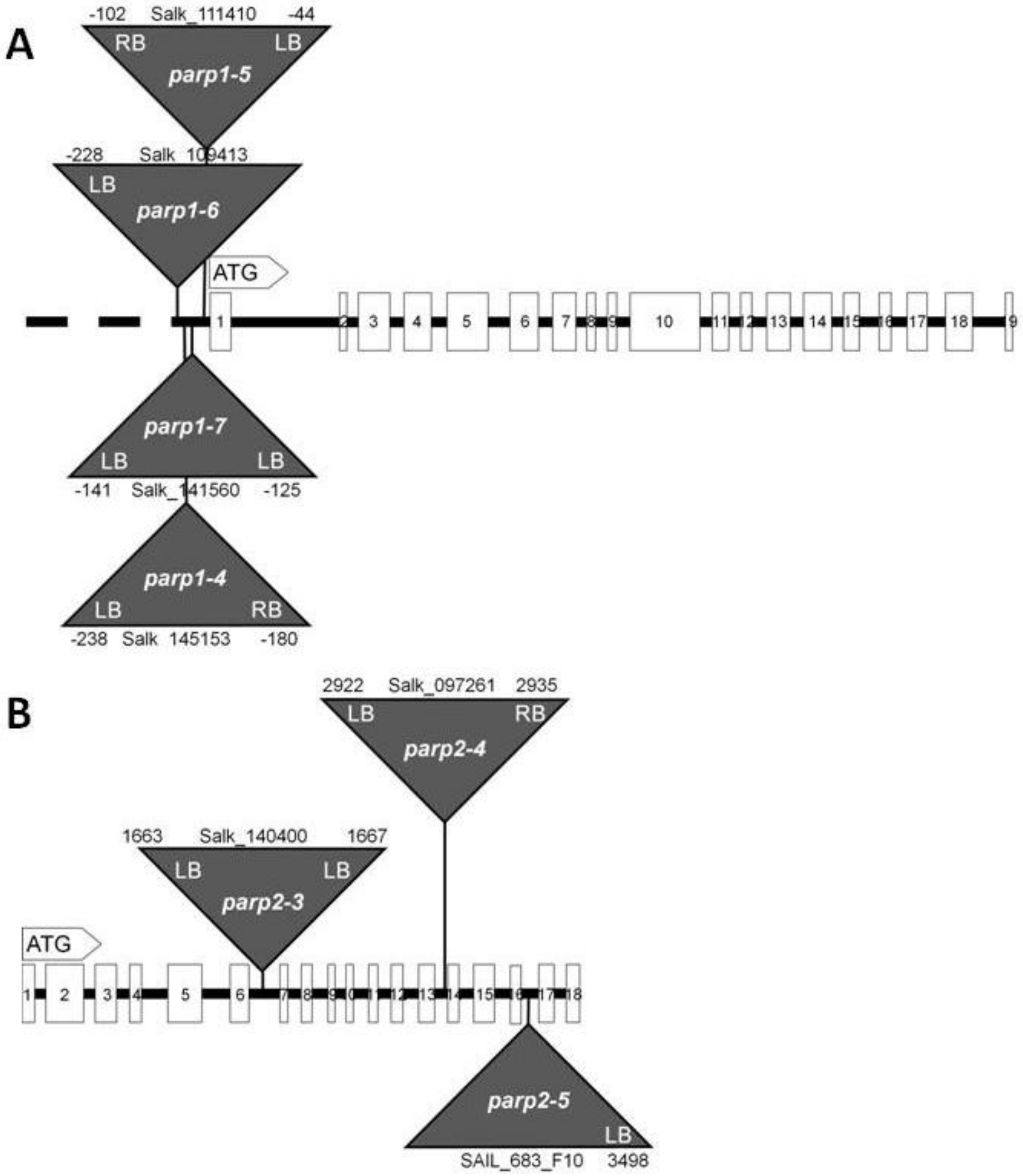

Supplementary Figure 1. Additional T-DNA insertion lines identified for *PARP1* and *PARP2*. Model of the genomic regions and the T-DNA insertions of *PARP1* (A) and *PARP2* (B). Coding regions are presented by white boxes, introns are shown by a line. Triangles indicate the sites of T-DNA insertion. The insertion lines originated from the SALK and the SAIL collections. The numbers indicate the last nucleotide before and the first nucleotide after the insertion, counting from the start codon. LB and RB indicate the left and right border of the T-DNA, as determined by sequencing.

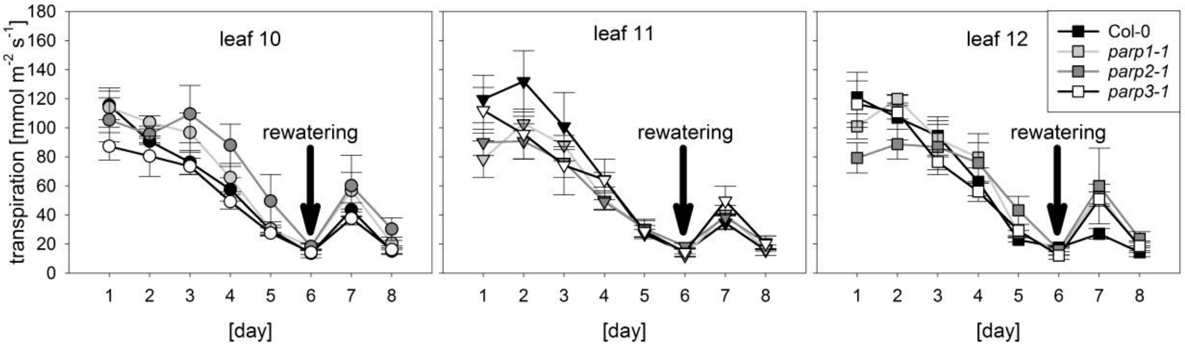

Supplementary Figure 2. Stomatal conductance is not altered in *parp* mutant plants compared to the wild type. Transpiration during desiccation was determined by porometry on leaves 10, 11, and 12. After 6 days plants were re-watered with 20 mL water. Data represent the means ±SE of 3-4 plants per line.

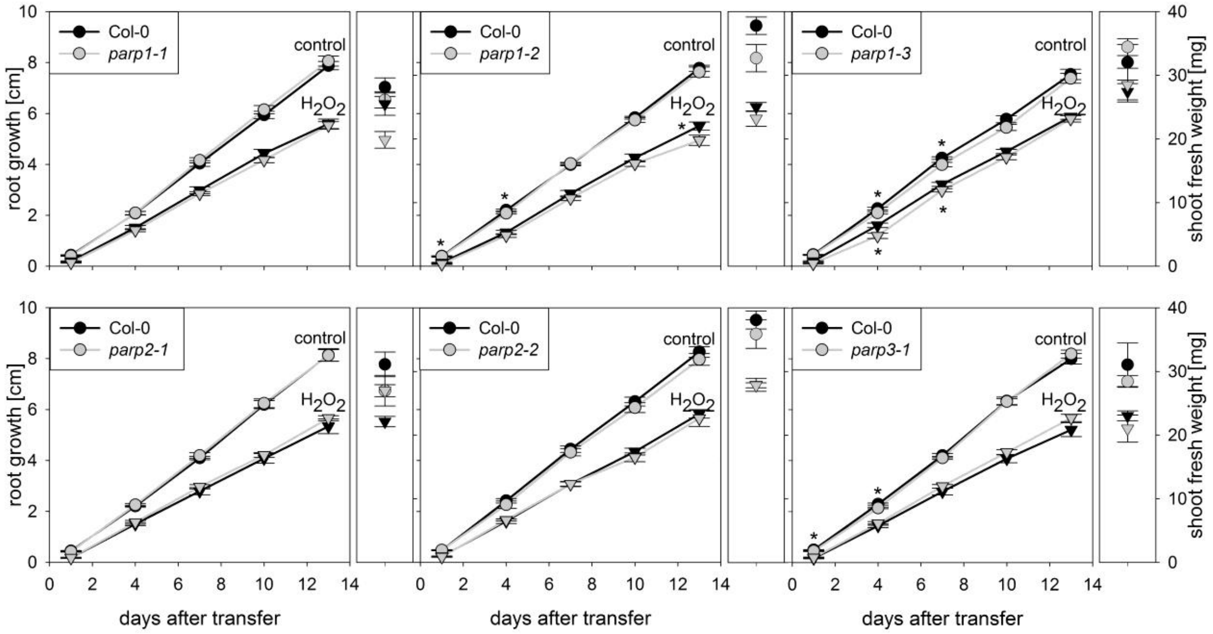

Supplementary Figure 3. Growth of *parp* mutant plants subjected to oxidative stress is not altered compared to the wild type. Root growth (large panels) of Col-0 and *parp* mutants on control plates (circles) or on plates containing 0.5 mM H_2O_2 (triangles). Shoot fresh weight was determined at the end of the experiment (small panels). The H_2O_2 treatment was contained in the experiment displayed in Figure 4. Control values shown in Figure 4 are included for comparison. Data represent the means ±SE of 15 plants per line.

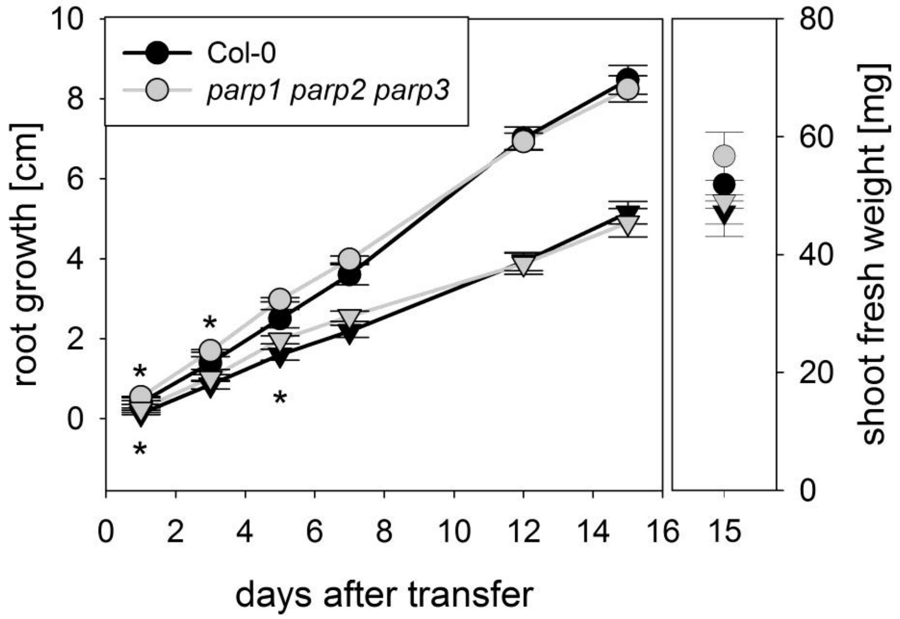

Supplementary Figure 4. Growth of *parp1-1 parp2-1 parp3-1* mutant plants subjected to oxidative stress is not altered compared to the wild type. Root growth (large panel) of Col-0 and *parp1-1 parp2-1 parp3-1* mutants on control plates (circles) or on plates containing 0.5 mM H_2O_2 (triangles). Shoot fresh weight was determined at the end of the experiment (small panel). The H_2O_2 treatment was contained in the experiment displayed in Figure 7. Control values shown in Figure 7 are included for comparison. Data represent the means ±SE of 15 plants per line.

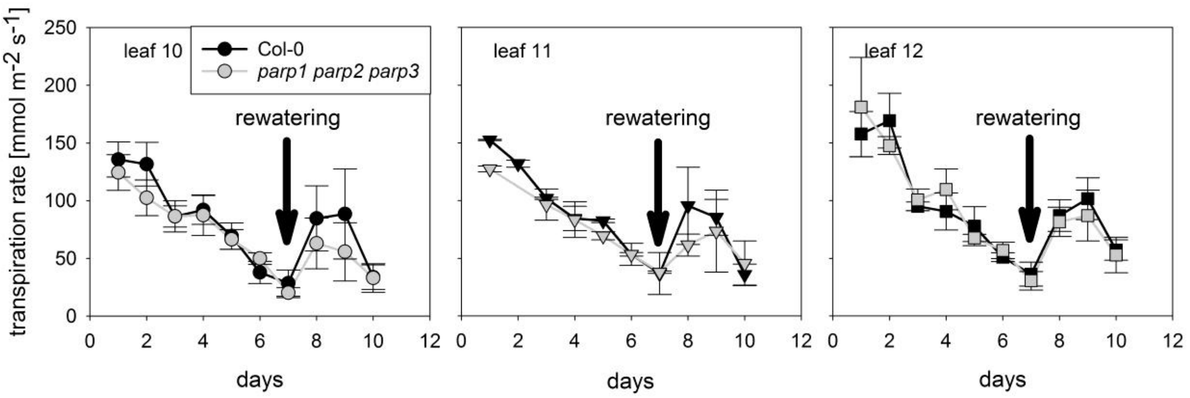

Supplementary Figure 5. Stomatal conductance is not altered in *parp1-1 parp2-1 parp3-1* mutant plants compared to the wild type. Transpiration during desiccation was determined by porometry on leaves 10, 11, and 12. After 7 days plants were re-watered with 20 mL water. Data represent the means ±SE of 3 plants per line.

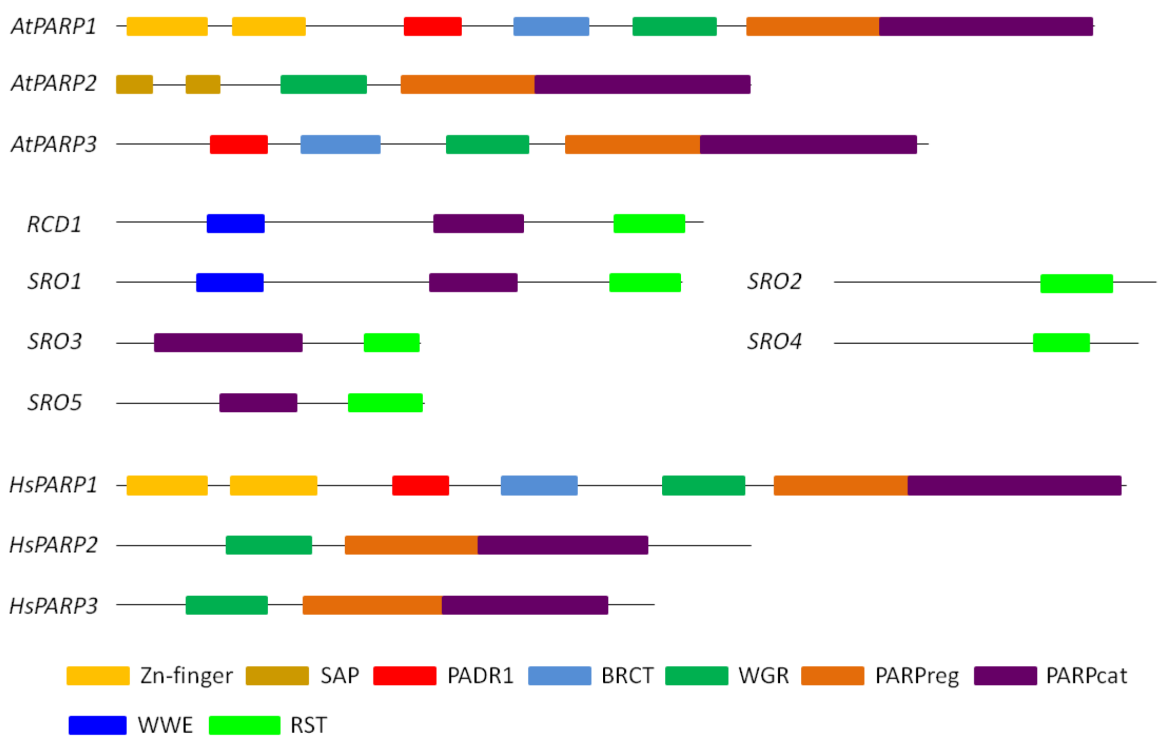

Supplementary Figure 6. Schematic representation of domains in animal and plant PARP proteins. Domains were defined according to Pfam 27.0 and are displayed as colored boxes. ExPASy Prosite indicated the existence of PARPcat domains also in SRO2 and SRO4, which are absent in the Pfam analysis.
